# Innate Immune Genes Associated With Newcastle Disease Virus Load in Chick Embryos From Inbred and Outbred Lines

**DOI:** 10.3389/fmicb.2019.01432

**Published:** 2019-06-20

**Authors:** Megan A. Schilling, Sahar Memari, Isabella M. Cattadori, Robab Katani, Amandus P. Muhairwa, Joram J. Buza, Vivek Kapur

**Affiliations:** ^1^Animal Science Department, Pennsylvania State University, University Park, PA, United States; ^2^Huck Institutes of the Life Sciences, Pennsylvania State University, University Park, PA, United States; ^3^School of Life Sciences and Bioengineering, The Nelson Mandela African Institution of Science and Technology, Arusha, TZ, United States; ^4^Applied Biological and Biosecurity Research Laboratory, Pennsylvania State University, University Park, PA, United States; ^5^Department of Biology, Pennsylvania State University, University Park, PA, United States; ^6^Department of Veterinary Medicine and Public Health, Sokoine University of Agriculture, Morogoro, TZ, United States

**Keywords:** Newcastle disease virus, innate immunity, chick embryo, Tanzanian ecotypes, susceptibility

## Abstract

Newcastle disease virus (NDV) causes substantial economic losses to smallholder farmers in low- and middle-income countries with high levels of morbidity and mortality in poultry flocks. Previous investigations have suggested differing levels of susceptibility to NDV between specific inbred lines and amongst breeds of chickens, however, the mechanisms contributing to this remain poorly understood. Studies have shown that some of these differences in levels of susceptibility to NDV infection may be accounted for by variability in the innate immune response amongst various breeds of poultry to NDV infection. Recent studies, in inbred Fayoumi and Leghorn lines, uncovered conserved, breed-dependent, and subline-dependent responses. To better understand the role of innate immune genes in engendering a protective immune response, we assessed the transcriptional responses to NDV of three highly outbred Tanzanian local chicken ecotypes, the Kuchi, the Morogoro Medium, and the Ching’wekwe. Hierarchical clustering and principal coordinate analysis of the gene expression profiles of 21-day old chick embryos infected with NDV clustered in an ecotype-dependent manner and was consistent with the relative viral loads for each of the three ecotypes. The Kuchi and Morogoro Medium exhibit significantly higher viral loads than the Ching’wekwe. The results show that the outbred ecotypes with increased levels of expression of CCL4, NOS2, and SOCS1 also had higher viral loads. The higher expression of SOCS1 is inconsistent with the expression in inbred lines. These differences may uncover new mechanisms or pathways in these populations that may have otherwise been overlooked when examining the response in highly inbred lines. Taken together, our findings provide insights on the specific conserved and differentially expressed innate immune-related genes involved the response of highly outbred chicken lines to NDV. This also suggests that several of the specific innate immunity related genes identified in the current investigation may serve as markers for the selection of chickens with reduced susceptibility to NDV.

## Introduction

Newcastle disease virus (NDV) is an important poultry pathogen in low- and middle-income countries where the disease is endemic (Ashraf and Shah, 2014). In these areas, poultry is farmed extensively with little to no veterinary care, minimal biosecurity, and the constant threat of exposure to pathogens (Minga et al., 2014; Muchadeyi and Dzomba, 2017). Vaccination and biosecurity in high-income countries and intensively farmed poultry control NDV, however, in low- to middle-income countries, it is uncommon or ineffective due to lack of proper storage of the vaccines or lack of adoption by smallholder farmers (Alexander et al., 2004, 2012; Wen et al., 2017; Campbell et al., 2018). Hence, there is a need for alternative strategies for the control of NDV in smallholder backyard traditional flocks.

Backyard farming heavily relies on natural selection to drive the adaptations and genetic diversity (Minga et al., 2014; Muchadeyi and Dzomba, 2017). These chickens are generally believed to be less susceptible to many poultry pathogens, however, there are few studies examining the genetic variation and disease resistance in attempt to explain their diversity and the distribution in Africa. One of the factors that may be influencing these mechanisms in response to NDV infection is the immune response. Until recently, most studies have focused on the adaptive or cell-mediated immune response of chickens to NDV due to the development of vaccines (Stone et al., 1997; Dimitrov et al., 2017; Wen et al., 2017), however, recent reports suggest the innate immune response has a major influence in the variability of the response to NDV infection (Rue et al., 2011; Schilling et al., 2018). These studies have demonstrated activation of the nitric oxide, the antiviral response, the interferon response and other important innate immune genes *in vitro*, *in vivo*, and *in ovo*, however, these mechanisms still remain poorly understood (Rue et al., 2011; Schilling et al., 2018).

We have previously described the use of the chick embryo, which becomes immunocompetent prior to hatch and in the protective environment of the shell, as a model to examine the innate immune response to NDV (Schilling et al., 2018, 2019). In one study, we found differential expression of innate immune genes from two highly inbred Fayoumi and Leghorn lines. The Fayoumi is less susceptible to many poultry pathogens, including *Salmonella*, Marek’s disease virus (MDV), and avian influenza virus (AIV). Recent studies have also reported a Fayoumi subline, M15.2, to be less susceptible to NDV than a Leghorn subline, Ghs6, through reduced viral shedding in lacrimal fluids post-NDV challenge. Our previous studies in these lines have uncovered breed-dependent differences in the level of susceptibility to NDV infection and innate immune genes that are differentially expressed in the response to NDV (Deist et al., 2017a, b, 2018; Schilling et al., 2018).

In Africa, the majority of poultry is farmed extensively and referred to as ecotypes (Minga et al., 2014). These ecotypes are named by smallholder farmers from different agro-ecological zones or farming regions and are based on phenotypic characteristics rather than genotypically distinct breeds (Minga et al., 2014). A study in Tanzania identified 17 local ecotypes based on a survey of local farmers in the country (Das et al., 2003). Three common Tanzanian ecotypes are the Morogoro medium, Kuchi, and Ching’wekwe. The Morogoro medium has multicolored plumage and widespread throughout Tanzania (Msoffe et al., 1998, 2001). The Kuchi is heaver and has a more upright stance compared to other ecotypes and mainly found in Northern Tanzania (Msoffe et al., 1998, 2001). The Ching’wekwe is a dwarf chicken with short legs and a small build (Msoffe et al., 1998, 2001). One study revealed the Kuchi may be resistant to *S. gallinarum* when compared to another local ecotype (Msoffe et al., 2001). Other studies also suggest the Tanzanian local ecotypes tend to cluster by ecotype according to DNA typing and microsatellite markers (Lyimo et al., 2013; Mwambene et al., 2019). There are studies suggesting differences in responsiveness to NDV vaccines in some Tanzanian local ecotypes, however, levels of susceptibility and mechanisms contributing to this remain unstudied (Lwelamira et al., 2009).

Given the breed-dependent differences uncovered in the response of highly inbred lines to NDV infection (Deist et al., 2017a, b, 2018; Zhang et al., 2018), we previously have shown that specific innate immune genes are driving this variation (Schilling et al., 2018, 2019). Through combining this with our previous studies that validate the use of the chick embryo as a model to examine the innate immune response (Schilling et al., 2018), we examined the expression of a set of innate immune genes in the chick embryos of three Tanzanian ecotypes in response to NDV infection.

## Materials and Methods

### Ethics Statement and Animal Use

The animal use protocol was approved by the Pennsylvania State University IACUC committee (Protocol No. 47175). All methods were performed in accordance with the relevant guidelines and regulations outlined in this protocol. Embryonated eggs from three Tanzanian local chicken ecotypes (Ching’wekwe, Kuchi, and Morogoro Medium) were provided by the Sokoine University of Agriculture (Morogoro, TZ, United States). Eggs were transported to the Nelson Mandela African Institution of Science and Technology (Arusha, TZ, United States) and incubated at 37.5°C, 55% humidity, rotating hourly. The eggs were temporarily removed from the incubators to candle for viability and inoculate with virus.

### Virus

The lentogenic LaSota strain of NDV was kindly provided by Dr. Siba Samal at the University of Maryland, College of Veterinary Medicine (College Park, MD, United States). Viral titrations were performed with the final titer of the undiluted viral suspension of 10^7^ 50% egg infectious dose (EID_50_)/mL. The viral suspension was stored at -80°C until further use.

### Embryonated Egg Inoculations and Tissue Harvest

Embryonated eggs from each subline were candled and airsacs were marked at 18 days of embryonic development. Small puncture holes were made in the eggs just above the airsac and 0.1 mL of the viral suspension was deposited directly to the allantoic fluid. The eggs were sealed with adhesive glue and placed back in the incubators until removal for tissue harvest. Controls (uninfected eggs) were treated similarly with 0.1 mL of sterile 1X phosphate buffered saline (PBS).

After 72 h (as close to hatch as possible), the eggs were removed from incubators and placed at 4°C for 3–4 h to avoid opening eggs with viable chick embryos. The chick embryos were removed from the eggs and washed with 1X PBS. The lung tissues were harvested and immediately stored at -80°C prior to further use.

### RNA Extraction

Lung tissues from 20 experimental and 10 control chick embryos from each ecotype were homogenized using a Bead Ruptor 24 (Omni International, Kennesaw, GA, United States). The lung tissue, 10–15 1.5 mm silica beads (Biospec Products, Bartlesville, OK, United States), 1 mL RLT lysis buffer (QIAGEN RNeasy kit; QIAGEN, Inc., Germantown, MD, United States) were placed in each tube, and the tissues were homogenized. 600 uL of homogenate was transferred to a clean microcentrifuge tube and centrifuged at 10,000 rpm for 2 min to remove any debris. The supernatant was transferred directly to the gDNA elimination column from the RNeasy Kit and manufacturer’s protocols were followed.

### cDNA Synthesis

cDNA synthesis was performed immediately following RNA extraction using the High Capacity cDNA Reverse Transcription Kit (Applied Biosystems, Carlsbad, CA, United States). The manufacturer protocols were followed using 2 μg of each respective RNA sample. cDNA was stored at -20°C until transported to the US.

The cDNA was packaged and sent to the Pennsylvania State University according to the United States Veterinary Permit for Importation and Transportation of Controlled Organisms and Vectors (U.S. Department of Agriculture, Animal and Plant Health Inspection Service, Veterinary Services) Permit no. 135537, along with clearance from the Center for Disease Control and US Fish and Wildlife Services. The shipment was also in accordance of the Animal Health and Export Certificate for Biologicals granted by the United Republic of Tanzania Ministry of Livestock and Fisheries, Permit No. VIC/AR/ZIS/5041.

### Multiplex RT-PCR

Viral load in the samples was quantified through amplifying the M-gene of NDV (primers: Ì+4100: 5′-AGT GAT GTG CTC GGA CCT TC-3′; Ì-4220: 5′-CCT GAG GAG AGG CAT TTG CTA-3′) (Wise et al., 2004). These reactions were performed using the PowerUp SYBR Green Master Mix (Applied Biosystems, Carlsbad, CA, United States) according to manufacturer recommendations. The concentration of virus in each sample was calculated through the use of the Standard Curve method and the copy number of virus in each sample was calculated. One Ching’wekwe and one Morogoro Medium sample in the experimental group were not positive for NDV, so these samples were excluded in the analysis.

Custom Taqman Gene Expression Assays (Thermo Fisher Scientific, Waltham, MA, United States) were ordered with different dyes according to Supplementary Table 1. These genes were selected based on previous studies that revealed a conserved response that included MX dynamin Like GTPase 1 (Mx1), Interferon Regulator Factor 1 (IRF1), Interferon Regulatory Factor 7 (IRF7), Signal Transducer and Activator of Transcription (STAT1), and Suppressor of Cytokine Signaling 1 (SOCS1) in inbred Fayoumi and Leghorn lines. As well as a breed-dependent response that is driven by Interferon Induced with Helicase C Domain 1 (IFIH1), Lipopolysaccharide Induced TNF Factor (LITAF), Nitric Oxide Synthase 2 (NOS2), and Toll-like Receptor 3 (TLR3) in the Fayoumis and Cathelicidin Antimicrobial Peptide (CAMP), C-C Motif Chemokine Ligand 4 (CCL4) and Interleukin-8 (IL8) in the Leghorns (Schilling et al., 2019). The bioinformatic team at Thermo Fisher examined the reactions to ensure no cross-reactivity of the assays in each respective reaction (all including Beta-Actin (ACTB) as the housekeeping gene). The datasets analyzed for this study can be found in the Pennsylvania State University Data Commons at https://doi.org/10.26208/1pq6-w685.

The PCR reactions were carried out according to manufacturer’s protocols and the TaqMan Multiplex Master Mix (Applied Biosystems, Carlsbad, CA, United States). Gene expression was analyzed using the △△Ct method comparing infected △Ct values (normalized with the Ct of B-actin, housekeeping gene) with the average of the control △Ct values (normalized with the Ct of B-actin, housekeeping gene).

### Data Analysis

The dataset from our previously published work that examined the innate immune gene expression in highly inbred Fayoumi and Leghorn lines (publicly available at: https://www.ncbi.nlm.nih.gov/bioproject/479417) was filtered to include only the set of genes in the current study.

Figures and analyses were performed with the statistical package R (R Core Team, 2017). Specifically, hierarchical clustering was performed using pvclust package (nboot = 1000, hclust.method = “complete,” dist.method = “maximum”) and visualized with ggtree (Suzuki and Shimodaira, 2015; Yu et al., 2017) to examine the clustering of the gene expression profiles both by target and by ecotype. The Venn Diagram was generated with the VennDiagram package (Chen, 2017) to show the genes that are differentially expressed over the controls in the three ecotypes and the PCoA plots and corresponding statistical analysis were generated with the Vegan package to examine clustering of the gene expression profiles and factors contributing to the clustering (Oksanen et al., 2017). The Spearman correlation coefficients and corresponding *p*-values were calculated using the psych package (Revelle, 2010).

## Results

### The Kuchi and Morogoro Medium Exhibit Significantly Higher Viral Loads Than the Ching’wekwe

**FIGURE 1:**
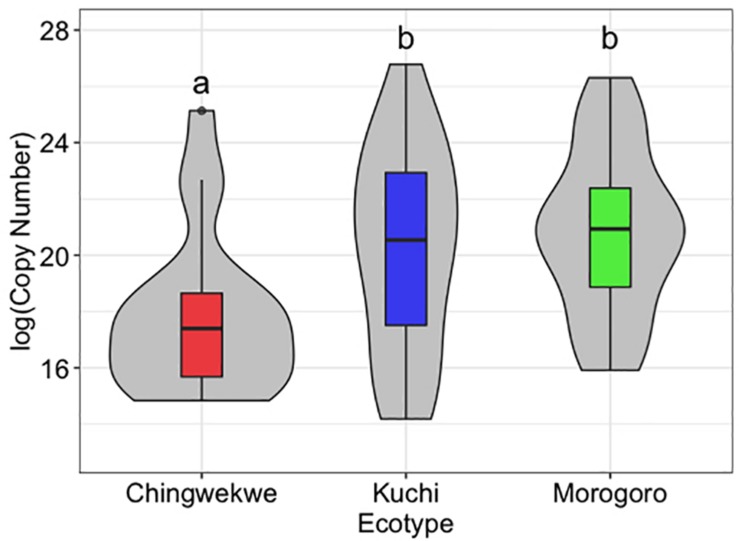
Viral load of Newcastle disease virus (NDV) in the chick embryo lung tissues. The viral load of NDV was assessed in the lung tissue of the chick embryos from each of the three ecotypes, with each ecotype along the *x*-axis and viral load [log(Copy Number)] on the *y*-axis. Within the boxplots, the middle line – median, box – Q1-Q3, whiskers – range, dots – outliers. The violin plots show the distribution of samples. The Ching’wekwe has a significantly lower viral load than the other two ecotypes (*p*-value < 0.05).

The Ching’wekwe had the lowest viral load, followed by the Kuchi (*p*-value compared to Ching’wekwe < 0.05), and the Morogoro Medium (*p*-value compared to Ching’wekwe < 0.01) (Figure 1). All controls were negative for the presence of NDV.

### The Innate Immune Genes Are Expressed Differentially Between the Three Ecotypes

**FIGURE 2:**
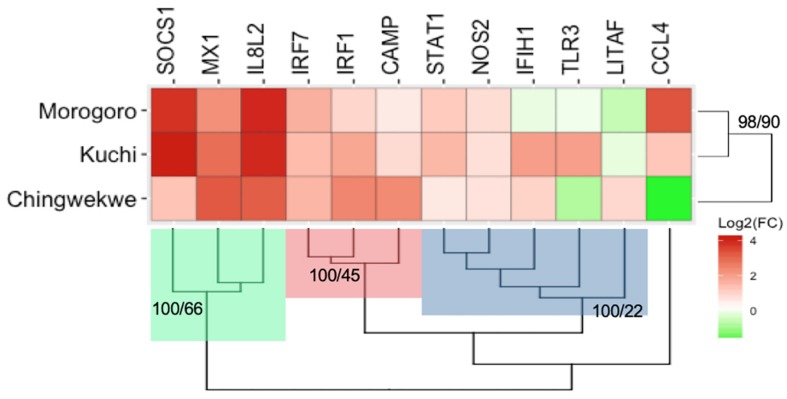
Heatmap and hierarchical clustering of innate immune gene expression profiles of the three Tanzanian ecotypes. The average log2(Fold Change) of the innate immune genes examined is illustrated in the heatmap. The red represents genes that are upregulated and the green represents genes that are downregulated. Hierarchical clustering was performed with the innate immune genes and the ecotypes. The targets clustered into three main groups, Cluster 1 (green), Cluster 2 (red), and cluster 3 (blue). The ecotypes group by the Morogoro Medium and Kuchi clustering together and with the Ching’wekwe as an outlier. The AU and BP values are shown at their respective nodes (all values are significant AU > 95).

The transcriptional response of a set of innate immune genes was examined in the lung tissues from the chick embryos of each ecotype using multiplex RT-PCR to examine if there are any differences in the response of the three ecotypes to NDV infection. The overall gene expression profiles, displaying the average expression of all genes, are illustrated in the heatmap in Figure 2 and the associated values are in Supplementary Table 2. The expression of the innate immune genes ranges from −3.6-fold of the CCL4 gene in the Ching’wekwe to 17.8-fold of the SOCS1 gene in the Kuchi. The majority of genes are upregulated in the response to NDV. Hierarchical clustering of the genes grouped the targets in three clusters: Cluster 1 (green) are all highly upregulated in the three ecotypes; Cluster 2 (red) are moderately upregulated in the three ecotypes while Cluster 3 (blue) are minimally upregulated to downregulated in the three ecotypes. The associated Approximately Unbiased (AU) and Bootstrap Probability (BP) are represented at the base node of each cluster (AU > 95 are statistically significant). The hierarchical clustering of the overall gene expression profiles by ecotype demonstrates that the Kuchi and Morogoro Medium gene expression profiles cluster together with the Ching’wekwe as an outlier (AU > 95) (Figure 2).

The average expression of the genes that are significantly and differentially expressed in the experimental group over the controls for each ecotype are shown in Supplementary Figure 1 (*p*-value < 0.1). The majority of genes are upregulated in response to NDV infection and TLR3 is downregulated in the Morogoro Medium. The Venn Diagram also demonstrate a conserved response between the three ecotypes that includes Mx1, IRF7, STAT1, and IL8 (Supplementary Figure 2). An ecotype-dependent response was also uncovered. The Ching’wekwe and Kuchi both have IFIH1 and IRF1 differentially expressed within the ecotype. Upregulation of both CAMP and NOS2 is unique to the Ching’wekwe, while an upregulation of TLR3 in the Kuchi, and an upregulation of CCL4 and SOCS1 and a downregulation of TLR3 are specific in the Morogoro Medium (Supplementary Figure 2).

### There Was an Ecotype-Dependent Clustering of the Transcriptional Innate Immune Gene Response to NDV Infection in the Highly Outbred Lines

**FIGURE 3:**
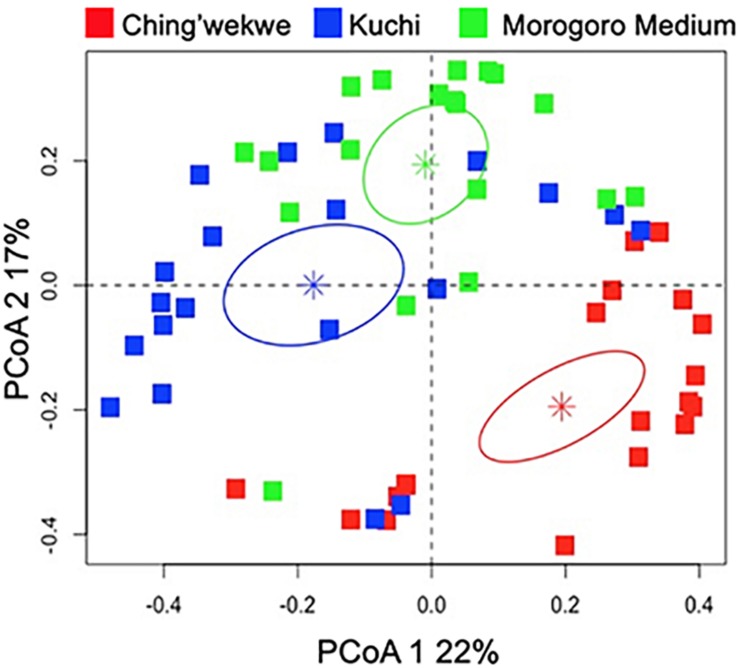
Principal coordinate analysis of the gene expression profiles of the three ecotypes. The PCoA plot demonstrates significant clustering of each ecotype independently (*p*-value < 0.01). The Ching’wekwe is represented by the red squares, the Kuchi by blue and Morogoro Medium by green. The stars represent the average for each ecotype and the ellipse is the 95% confidence interval (in each respective color).

**FIGURE 4:**
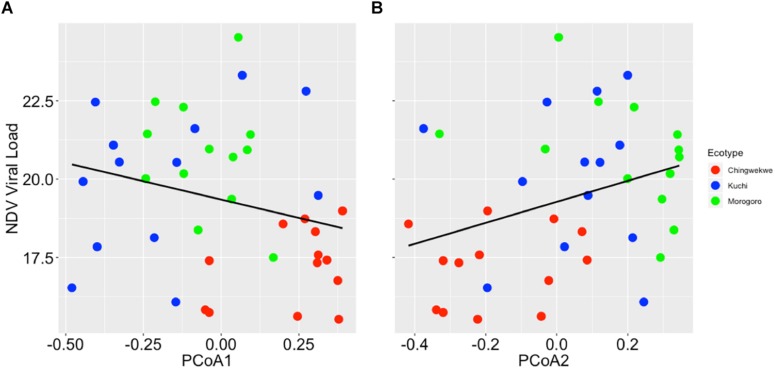
Relationship between principal coordinate (PCoA) axis and NDV viral load. **(A)** The NDV viral load versus the PCoA axis 1 (0.30 ± 0.26, *p*-value = 0.05). The expression of NOS2 is most representative for PCoA axis 1. **(B)** The NDV viral load versus the PCoA axis 2 (0.30 ± 0.30, *p*-value = 0.05). The expression of CCL4, CAMP, and IL8 are most representative for PCoA axis 2.

To further investigate the differences in the gene expression profiles of the three ecotypes, principal coordinate analysis was performed to examine the grouping of the overall gene expression profiles. The results showed that the gene expression profiles cluster in an ecotype-dependent manner (*p*-value < 0.01, Figure 3) that is consistent with the hierarchical clustering and viral load. For instance, the distances between the averages (represented by the colored asterisks in Figure 3) are closer between the Kuchi and Morogoro Medium than with the Ching’wekwe as was earlier noted (Figure 2). To assess the primary drivers of the differences between the grouping, the results show that the first two principal coordinates of the PCoA account for 39% of the total variation. While it in not unexpected that only a relatively small fraction of the total variation might be explained in highly outbred lines, the analysis did provide evidence of individual genes that influenced the grouping. For example, the results show that the first principal coordinate was driven by the effect of the expression of NOS2 (coefficient = −0.86), and the second principal coordinate is affected by the expression of CAMP (−0.99), IL8 (−0.87), and CCL4 (−0.90). When differences in viral load were considered in relation to the estimated principal coordinates, the results show no significant relationship between the first and second principal coordinates when using the entire dataset (Spearman correlation coefficient – PCoA1: −0.13, *p*-value = 0.17; PCoA2: 0.16, *p*-value = 0.11, Supplementary Figure 3). However, if the outliers were excluded and only the viral load data ranging between the first and third quartiles (Supplementary Table 3) were considered to reduce the within ecotype variability, of each ecotype, a significant relationship emerges (PCoA1: −0.27, *p*-value = 0.05; PCoA2: 0.33, *p*-value = 0.02, Figure 4) that is suggestive of a negative relationship between PCoA axis 1 and viral load and positive relationship between PCoA axis 2 and viral load (Supplementary Figure 4). To explore the secondary relationships of the PCoA axes and differences in viral load, the influence of individual genes driving the observed groupings were examined. The results suggest no significant relationship between NOS2 and viral load (0.15, *p*-value = 0.13), which is expected since NOS2 is seen to drive the grouping of PCoA1. However, the results reveal a positive association between viral load and the expression of CCL4 (0.43, *p*-value < 0.01) and IL8 (0.61, *p*-value < 0.01); and a negative association between viral load and the expression of CAMP (−0.42, *p*-value < 0.01). Taken together, the results are suggestive of a general trend that viral load tends to increase with the expression of CCL4 and IL8 and decreases with expression of CAMP.

Further, the relationships between the expression of these four genes and viral load were also examined within each ecotype (Supplementary Figure 5). The results show a negative relationship between the expression of NOS2 and viral load (−0.51, *p*-value < 0.05) in the Ching’wekwe In contrast, there were strong positive relationships between the expression of both CCL4 (0.66, *p*-value < 0.01) and IL8 (0.66, *p*-value < 0.01) with viral load within the Kuchi ecotype. Finally, the analyses revealed a positive relationship between the expression of NOS2 and viral load in the Morogoro Medium (0.58, *p*-value < 0.05).

### There Was Breed-Dependent Clustering of the Transcriptional Innate Immune Gene Response to NDV Infection in Highly Inbred Lines

**FIGURE 5:**
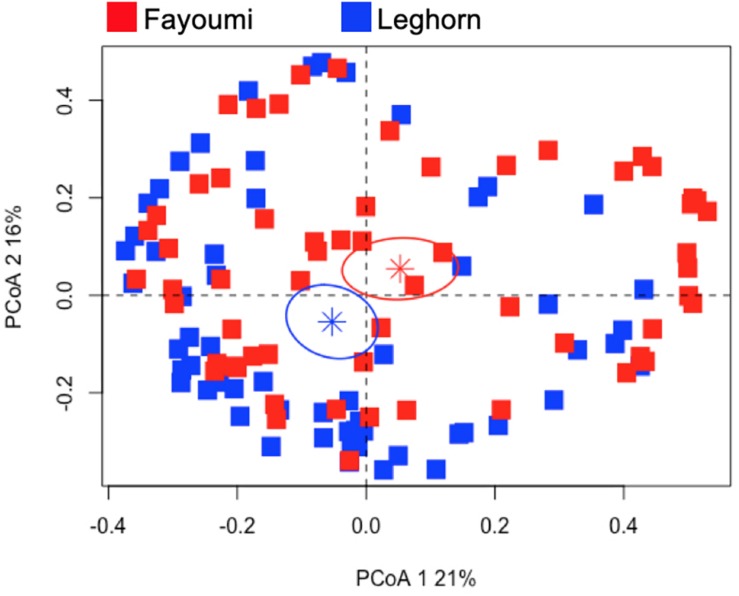
Principal coordinate analysis of the gene expression profiles of the Fayoumi and Leghorn. The gene expression profiles from the previous study were filtered to only include the same genes examined in the outbred lines. The PCoA plot demonstrates clustering of each breed (*p*-value < 0.01). The Fayoumi is represented by the red squares and the Leghorn by. The stars represent the average for each breed and the ellipse is the 95% confidence interval (in each respective color).

Since the selection of this set of genes was from a previous study in highly inbred Fayoumi and Leghorn lines, we examined the dataset from our previously published work to examine if similar genes are influencing the clustering of those lines. We filtered the dataset for only the set of genes in the current study to investigate the breed-dependent response. The PCoA applied to this dataset shows the distinct clustering of two breeds (*p*-value < 0.01, Figure 5) that also differ in their viral load and level of susceptibility to NDV (Supplementary Figure 6). The first two principle coordinates explain 37% of the variation in these samples. The first principle coordinate was driven by the effect of the expression of CCL4 (0.80), IFIH1 (0.99), IL8 (0.95), IRF1 (0.92), IRF7 (0.88), LITAF (0.99), MX1 (0.94), NOS2 (0.96), STAT1 (0.96), and TLR3 (0.99). The second principle coordinate was affected by the expression CAMP (0.86) and CCL4 (0.60).

### A Few Innate Immune Genes Are Similarly Differentially Expressed in Both Highly Inbred and Highly Outbred Lines

**FIGURE 6:**
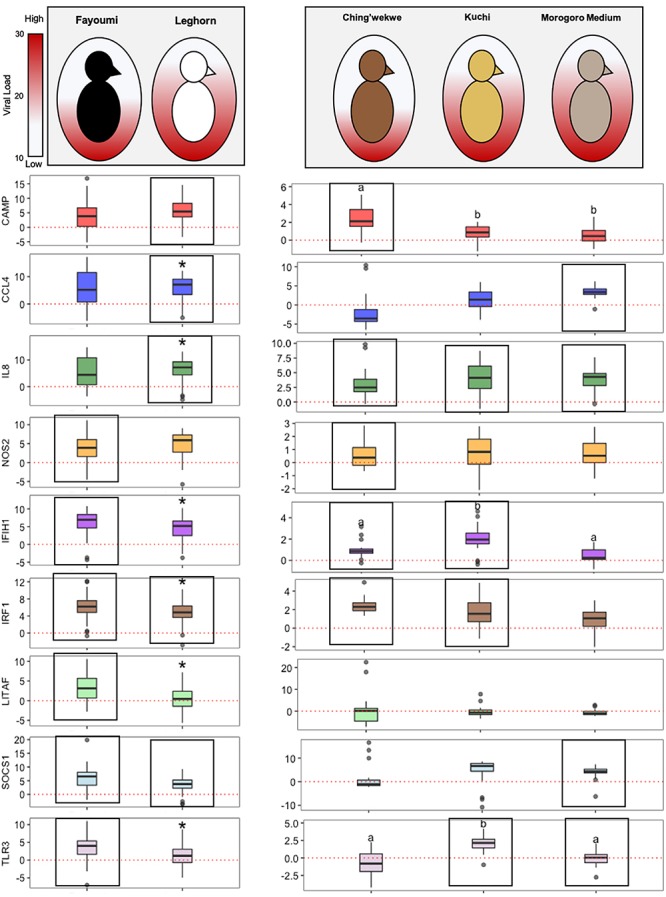
Overall breed- and ecotype-dependent innate immune gene expression. The inbred lines, Fayoumi and Leghorn are shown on the left side of the graph and the outbred lines, Tanzanian ecotypes, are on the right. The shading in each egg represents the average viral load of each line (the more color, the higher the viral load). The boxplots show the log2(Fold Change) of each gene (middle line – median, box – Q1–Q3, whiskers – range, dots – outliers). The red line in each graph is at a y-intercept of 0, anything above the line is upregulated and anything below is downregulated. The statistical significance is represented by the ^*^ in the inbred and letters in the outbred graphs (*p*-value < 0.05). The boxes outline the targets that are statistically differentially expressed in the experimental group (infected) versus control group (uninfected) in each line.

Through examining the statistically and differentially expressed innate immune genes in both the inbred and outbred lines, a few are associated with the response of the different lines to NDV infection (Figure 6). CCL4 is differentially expressed in the Leghorn and Morogoro Medium, which both have higher viral loads when compared to the other lines. On the other hand, NOS2 is differentially expressed in the Fayoumi and Ching’wekwe, which both have lower viral loads.

### Some Genes Are Regulated Differently Between the Highly Inbred and Highly Outbred Lines

Inbred and outbred lines showed a different ability to regulate genes, particularly for CAMP, SOCS1, IL8, and IRF1, in the response to NDV infection (Figure 6). CAMP is differentially upregulated in the Ching’wekwe and Leghorn lines which differ in their relative viral loads. SOCS1 is differentially regulated in both inbred lines, however, only differentially upregulated in the Morogoro Medium. This is opposite in IL8, which is differentially upregulated in all three ecotypes, however only differentially upregulated in the Leghorn. IRF1 is differentially expressed over the controls in all lines except the Morogoro Medium.

## Discussion

Understanding the genes and mechanisms influencing the response to NDV, particularly in backyard chicken populations, will provide a framework for genetic improvement of poultry through determining markers for reduced susceptibility to NDV. Our study began to specify genes that are influencing the variation observed in the level of susceptibility of both inbred and outbred lines to NDV infection.

Studies examining the level of susceptibility to many pathogens, including NDV, have used phenotypic traits such as viral load, antibody response, weight, morbidity, and mortality (Liu et al., 2003; Wigley et al., 2006; Deist et al., 2017b). However, since we focused on chick embryos rather than hatched chicks, we used viral load as our estimator to determine the level of susceptibility of the different lines to NDV infection as it is a direct measure of the ability of the virus to replicate in a permissive system. Although this might be a limitation to our study and other assays, such as a viral plaque assay, may have provided better information regarding infectious virus particles, we are able to demonstrate differences in the viral load of Tanzanian ecotypes through qPCR-based methods. The viral load was ascertained in the lung tissue of the chick embryos 72 h post-infection, when the viral suspension was deposited directly into the allantoic fluid. Since there were relatively high viral loads in the lungs of the chick embryos from the different lines, this suggests the chick embryos were likely infected. Here, we showed that the Ching’wekwe (lowest viral load) was the least susceptible to NDV, followed by Kuchi (moderate viral load), then Morogoro Medium (high viral load) which was the most susceptible to NDV (Figure 2). Until now, many studies have hinted that certain ecotypes are more or less susceptible to certain pathogens, including NDV (Msoffe et al., 1998, 2001; Lyimo et al., 2013; Minga et al., 2014). However, now we provided evidence that these claims are true, although further supporting these results through studies examining the level of susceptibility in hatched chicks are needed to fully understand the level of susceptibility to NDV in the outbred ecotypes.

The transcriptional profiles of the innate immune genes in chick embryos from different breeds and ecotypes are clustering in an ecotype-dependent or breed-dependent manner. The clustering patterns using both the hierarchical clustering and PCoA are consistent by showing that the Kuchi and Morogoro Medium are more closely related with each other than the Ching’wekwe (Figures 1, 3). Consistent with previous studies examining genetic variations, using DNA typing and microsatellite markers, in these ecotypes, clustering was also ecotype-dependent (Msoffe et al., 1998; Lyimo et al., 2013; Mwambene et al., 2019).

Through examining specific innate immune genes, there are striking differences in expression patterns between susceptible versus resistant lines as well as between the inbred and outbred lines. Some targets stand out as being differentially expressed between breeds and ecotypes, including CCL4 and NOS2. Another set of genes, expressed differently in the inbred versus outbred lines, includes CAMP, SOCS1, IL8, and IRF1. Studies examining these genes and genomic regions more thoroughly may reveal important mechanisms associated with susceptibility to NDV infection.

An interesting gene that was differentially expressed in the susceptible lines is CCL4, which is influencing susceptibility to NDV. In this study, overall CCL4 was positively correlated with viral load in the ecotypes (Supplementary Figure 4), which was also evident in the average expression between the ecotypes with CCL4 being downregulated in the Ching’wekwe and upregulated in the Morogoro Medium. More specifically, CCL4 is positively correlated with the viral load in the Kuchi ecotype. CCL4 is a key pro-inflammatory cytokine that is important in the innate immune response and the clearance of virus (Withanage et al., 2005). A previous study in two White Leghorn lines differing in their levels of susceptibility to Necrotic Enteritis lines demonstrated that CCL4 was also differentially upregulated in the susceptible versus resistant line (Truong et al., 2015).

Another gene, NOS2, is significantly differentially expressed in resistant lines rather than the susceptible lines, which may influence resistance to NDV. However, the expression of NOS2 is negatively associated with viral load in the Ching’wekwe ecotype, but positively correlated with the Morogoro Medium. This is interesting since high viral load in the resistant line is associated with lower fold changes of NOS2, but on the other hand, in the susceptible line this is opposite and high viral load is associated with higher fold changes in gene expression. NOS2 is commonly upregulated in the response of many chicken lines to different infections. NOS2 is responsible for the induction of nitric oxide (NO), a free radical linked to multiple different immune responses, particularly in the clearance of viruses (Kapczynski et al., 2013). A previous study examined the expression of NOS2 in a high responder line versus a low responder line to *Salmonella* infection (Kapczynski et al., 2013). In this study, the high responder line demonstrated differential expression of NOS2 than the low responder line (Singh et al., 2012). However, it is important to note that the induction of NO at high levels may cause more harm than good to the host (Burggraaf et al., 2011; Rue et al., 2011).

Curiously, CAMP, which was negatively correlated with viral load (Supplementary Figure 4), was responding differently in the inbred versus outbred lines; it was significantly differentially regulated in the Ching’wekwe and in the Leghorn. CAMP has a broad spectrum of antimicrobial activity particularly against bacteria, fungi, and enveloped viruses in all organisms (Kościuczuk et al., 2012). It is expressed in various cell types and is related to the defensins and TLR pathways (Kościuczuk et al., 2012; Jeon et al., 2016). Although many studies have demonstrated the antimicrobial effects of CAMP, mechanistically, much remains unstudied. This gene, particularly due to the differences between the inbred and outbred lines is a strong candidate for future studies to understand how the expression of CAMP plays a role in the response to NDV infection.

Another gene expressed differently between the inbred and outbred lines is SOCS1. In the inbred lines, SOCS1 was a part of the conserved response, differentially upregulated in both breeds, however, in the outbred lines it was only differentially regulated in the Morogoro Medium. SOCS1 is a negative regulator of cytokine functioning and reduces interferon signaling pathways (Giotis et al., 2017). This displays how SOCS1, as well, may play an important role in the response of outbred lines to NDV since it is downregulated in the resistant line and upregulated in susceptible lines.

In the inbred lines, IRF1 was a part of the conserved response to NDV, and IL8 was in the conserved response of the outbred lines. The expression of IL8 in the Fayoumi was just below the cutoff for significance, demonstrating this gene may be a part of the conserved response of all lines and important in the response to NDV regardless of breed or ecotype. Although IRF1 may not be as close statistically, it may still be an important gene in the response to NDV regardless of line.

Through examining these genes that are important in the response to NDV infection in the different lines, it is interesting to note that all of these genes (with the exception of CAMP) are negatively selected for when related to disease resistance, particularly in the outbred lines. High expression of CCL4, NOS2, and SOCS1 have negative effects related to susceptibility to NDV. This poses two interesting hypotheses: (1) The basal levels of innate immune gene expression are higher in resistant lines, contributing to lower fold changes upon infection. Or (2) The resistant lines have adapted so that upon infection, since these pathogens are encountered often in the field, the innate immune response does not overreact and cause greater harm in the organism. Understanding the mechanisms and pathways these genes, and particularly SOCS1, are involved in may uncover new information in these populations that may have otherwise been overlooked when examining the response in highly inbred lines.

Taking these data together, we are able to propose a set of genes that are differentially regulated, either dependent on the level of susceptibility to NDV or between inbred and outbred lines, in the response of different chicken lines to NDV infection. These genes and genomic regions may serve as possible candidates for future studies on resistance to NDV to determine markers for breeding strategies to improve disease resistance. Expanding these studies to include other tissues from the chick embryos, and possibly other timepoints of tissue harvest, more outbred lines and different pathogens, as well as associating these studies with the level of susceptibility and the immune response in hatched chicks are needed to fully understand the innate immune response in chickens. This study and future studies on these candidate genes and regions will provide a framework to determine markers for resistance or susceptibility to NDV that could be used to enhance breeding strategies and improve yields for smallholder farmer, especially in low- to middle-income countries.

## Data Availability

All datasets generated for this study are included in the manuscript and/or the Supplementary Files.

## Author Contributions

MS, VK, and JB designed the study. MS and SM performed laboratory experiments. MS performed the data analysis and wrote the manuscript. IC guided the statistical analysis. IC, RK, AM, JB, and VK critically revised the content. All authors read and approved the final manuscript.

## Conflict of Interest Statement

The authors declare that the research was conducted in the absence of any commercial or financial relationships that could be construed as a potential conflict of interest.

## References

[B1] Alexander D. Bell J. Alsers R. (2004). *A Technology Review: Newcastle Disease - With Special Emphasis on its Effects on Village Chickens.* Rome: Food and Agriculture Organization of the United Nations.

[B2] Alexander D. J. Aldous E. W. Fuller C. M. (2012). The long view: a selective review of 40 years of newcastle disease research. *Avian Pathol.* 41 329–335. 10.1080/03079457.2012.697991 22834545

[B3] Ashraf A. Shah M. (2014). Newcastle disease: present status and future challenges for developing countries. *Afr. J. Microbiol Res* 8 411–416. 10.5897/AJMR2013.6540

[B4] Burggraaf S. Bingham J. Payne J. Kimpton W. G. Lowenthal J. W. Bean A. G. D. (2011). Increased inducible nitric oxide synthase expression in organs is associated with a higher severity of H5N1 influenza virus infection. *PLoS One* 6:e14561. 10.1371/journal.pone.0014561 21283521 PMC3023712

[B5] Campbell Z. A. Marsh T. L. Mpolya E. A. Thumbi S. M. Palmer G. H. (2018). Newcastle disease vaccine adoption by smallholder households in Tanzania: identifiying determinants and barriers. *PLoS One* 13:e0206058. 10.1371/journal.pone.0206058 30356260 PMC6200240

[B6] Chen H. (2017). *VennDiagram: Generate High-Resolution Venn and Euler plots. R Package Version 1.6.18.*

[B7] Das S. M. Msechu J. K. K. Kyomo M. L. Mpiri D. B. Chenyambuga S. W. (2003). “State of farm animal genetic resources in Tanzania,” in *Proceedings of the 20th Tanzania Veterinary Association Veterinary Conference*, Tanzania.

[B8] Deist M. S. Gallardo R. A. Bunn D. A. Dekkers J. C. Zhou H. Lamont S. J. (2017a). Resistant and susceptible chicken lines show distinctive responses to newcastle disease virus infection in the lung transcriptome. *BMC Genomics* 18:989. 10.1186/s12864-017-4380-4384 29281979 PMC5745900

[B9] Deist M. S. Gallardo R. A. Bunn D. A. Kelly T. R. Dekkers J. C. Zhou H. (2017b). Novel mechanisms revealed in the trachea transcriptome of resistant and susceptible chicken lines following infection with Newcastle disease virus. *Clin. Vaccine Immunol.* 24:e027-17. 10.1128/CVI.00027-17 28331077 PMC5424241

[B10] Deist M. S. Gallardo R. A. Bunn D. A. Kelly T. R. Dekkers J. C. Zhou H. (2018). Novel analysis of the harderian gland transcrptome response to newcastle disease virus in two inbred chicken lines. *Sci. Rep.* 8:6558. 10.1038/s41598-018-24830-24830 29700338 PMC5920083

[B11] Dimitrov K. M. Afonso C. L. Yu Q. Miller P. J. (2017). Newcastle disease vaccines - a solved problem or a continuous challenge? *Vet. Microbiol.* 206 126–136. 10.1016/j.vetmic.2016.12.019 28024856 PMC7131810

[B12] Giotis E. S. Ross C. S. Robey R. C. Nohturfft A. Goodbourn S. Skinner M. A. (2017). Constitutively elevated levels of SOCS1 suppress innate responses in DF-1 immortalised chicken fibroblast cells. *Sci. Rep.* 7:17485. 10.1038/s41598-017-17730-17732 29235573 PMC5727488

[B13] Jeon I.-S. Kim J. S. Jeon M.-H. Byun S. J. Lee S. I. Jang H. J. (2016). Transcriptional regulation of cathelicidin genes in chicken bone marrow cells. *Poult. Sci.* 95 912–919. 10.3382/ps/pev361 26908883

[B14] Kapczynski D. R. Afonso C. L. Miller P. J. (2013). Immune responses of poultry to newcastle disease virus. *Dev. Comp. Immunol.* 41 447–453. 10.1016/j.dci.2013.04.012 23623955

[B15] Kościuczuk E. M. Lisowski P. Jarczak J. Strzałkowska N. Jóźwik A. Horbańczuk J. (2012). Cathelicidins: family of antimicrobial peptides. a review. *Mol. Biol. Rep.* 39 10957–10970. 10.1007/s11033-012-1997-x 23065264 PMC3487008

[B16] Liu W. Kaiser M. G. Lamont S. J. (2003). Natural resistance-associated macrophage protein 1 gene polymorphisms and response to vaccine against or challenge with *Salmonella* enteritidis in young chicks. *Poult. Sci* 82 259–266. 10.1093/ps/82.2.259 12619803

[B17] Lwelamira J. Kifaro G. C. Gwakisa P. S. (2009). Genetic parameters for body weights, egg traits and antibody response against newcastle disease virus (NDV) vaccine among two Tanzania chicken ecotypes. *Trop. Anim. Health Prod.* 41 51–59. 10.1007/s11250-008-9153-9152 19052902

[B18] Lyimo C. M. Weigend A. Janßien-Tapken U. Msoffe P. L. Simianer H. Weigend S. (2013). Assessing the genetic diversity of five Tanzanian chicken ecotypes using molecular tools. *S. Afr. J. Anim. Sci.* 43 499–510.

[B19] Minga U. M. Msoffe P. Gwakisa P. (2014). “Biodiversity (variation) in disease resistance and in pathogens within rural chicken populations,” in *Proceedings of the 22nd World’s Poultry Conferences*, Istanbul.

[B20] Msoffe P. Mtambo M. Minga U. M. Gwakisa P. Katule A. M. Mutayoba S. K. (1998). Scavenging local chickens in Tanzania: is there room for improvement? *Tanzanian Vet. J.* 18(Suppl. 4), 117–126.

[B21] Msoffe P. Mtambo M. Minga U. M. Gwakisa P. Mdegala R. Olsen J. E. (2001). Phenotypes including immunocompetence in scavenging local chickens in Tanzania. *Trop. Anim. Health Prod.* 33 341–354. 11474868 10.1023/a:1010544221028

[B22] Muchadeyi F. C. Dzomba E. F. (2017). *Genomics Tools for the Characterization of Genetic Adaptation of Low Input Extensively Raised Chickens.* London: IntechOpen.

[B23] Mwambene P. L. Githae D. Machuka E. Kyallo M. Pelle R. (2019). Genetic diversity of 10 indigenous chicken ecotypes from southern highlands of Tanzania based on major histocompatibility complex-linked microsatellite LEI0258 marker typing. *Poult. Sci.* 10.3382/ps/pez076 [Epub ahead of print]. 30877744 PMC6591683

[B24] Oksanen J. Blanchet F. G. Friendly M. Kindt R. Legendre P. McGlinn D. (2017). *vegan: Community Ecology package. R package version 2.4–5.*

[B25] R Core Team (2017). *R: A Language and Environment for Statistical Computing.* Vienna: R Foundation for Statistical Computing.

[B26] Revelle W. (2010). *Package “psych” Version 1.0-92.*

[B27] Rue C. A. Susta L. Cornax I. Brown C. C. Kapczynski D. R. Suarez D. L. (2011). Virulent newcastle disease virus elicits a strong innate immune response in chickens. *J. Gen. Virol.* 92 931–939. 10.1099/vir.0.025486-0 21177922

[B28] Schilling M. A. Katani R. Memari S. Cavanaugh M. Buza J. J. Radzio-Basu J. (2018). Transcriptional innate immune response of the developing chicken embryo to Newcastle disease virus infection. *Front. Genet.* 9:61. 10.3389/fgene.2018.00061 29535762 PMC5835104

[B29] Schilling M. A. Memari S. Cavanaugh M. Katani R. Deist M. S. Radzio-Basu J. (2019). Conserved, breed-dependent, and subline-dependent innate immune responses of fayoumi and leghorn chicken embryos to Newcastle disease virus infection. *Sci. Rep.* 9:7209. 10.1038/s41598-019-43483-43481 31076577 PMC6510893

[B30] Singh R. Jain P. Pandey N. K. Saxena V. K. Saxena M. Singh K. B. (2012). Cytokines expression and nitric oxide production under induced infection to *Salmonella* Typhimurium in chicken lines divergently selected for cutaneous hypersensitivity. *Asian Aust. J. Anim. Sci.* 25 1038–1044. 10.5713/ajas.2011.11324 25049661 PMC4092978

[B31] Stone H. Mitchell B. Brugh M. (1997). In ovo vaccination of chick embryos with experimental newcastle disease and avian influenza oil-emulsion vaccines. *Avian Dis.* 41 856–863. 9454919

[B32] Suzuki R. Shimodaira H. (2015). *pvclust: Hierarchical Clustering With p-Values via Multiscale Bootstrap Resampling. R Package Version 2.0–0.*

[B33] Truong A. D. Hong Y. H. Lillehoj H. S. (2015). RNA-seq profiles of immune related genes in the spleen of necrotic enteritis-afflicted chicken lines. *Asian Aust. J. Anim. Sci.* 28 1496–1511. 10.5713/ajas.15.0143 26323406 PMC4554858

[B34] Wen G. Li L. Yu Q. Wang H. Luo Q. Zhang T. (2017). Evaluation of a thermostable newcastle disease virus strain TS09-C as an in-ovo vaccine for chickens. *PLoS One* 12:e0172812. 10.1371/journal.pone.0172812 28234989 PMC5325573

[B35] Wigley P. Hulme S. Rothwell L. Bumstead N. Kaiser P. Barrow P. (2006). Macrophages isolated from chickens genetically resistant or susceptible to systemic salmonellosis show magnitudinal and temporal differential expression of cytokines and chemokines following *Salmonella enterica* challenge. *Infect. Immun.* 74 1425–1430. 10.1128/IAI.74.2.1425-1430.2006 16428798 PMC1360331

[B36] Wise M. G. Suarez D. L. Seal B. S. Pedersen J. C. Senne D. A. King D. J. (2004). Development of a real-time reverse-transcription PCR for detection of newcastle disease virus RNA in clinical samples. *J. Clin. Microbiol.* 42 329–338. 10.1128/JCM.42.1.329-338.2004 14715773 PMC321685

[B37] Withanage G. S. K. Wigley P. Kaiser P. Mastroeni P. Brooks H. Powers C. (2005). Cytokine and chemokine responses associated with clearance of a primary *Salmonella enterica* serovar Typhimurium infection in the chicken and in protective immunity to rechallenge. *Infect. Immun.* 73 5173–5182. 10.1128/IAI.73.8.5173-5182.2005 16041035 PMC1201213

[B38] Yu G. Smith D. Zhu H. Guan Y. Lam T. T.-Y. (2017). ggtree: an R package for visualization and annotation of phylogenetic trees with thier covariates and other associated data. *Methods Ecol. Evol.* 8 28–36. 10.1111/2041-210x.12628

[B39] Zhang J. Kaiser M. G. Deist M. S. Gallardo R. A. Bunn D. A. Kelly T. R. (2018). Transcriptome analysis in spleen reveals differential regulation of response to newcastle disease virus in two chicken lines. *Sci. Rep.* 8:1278. 10.1038/s41598-018-19754-19758 29352240 PMC5775430

