# Supplementary material for: Innate Immune Genes Associated With Newcastle Disease Virus Load in Chick Embryos From Inbred and Outbred Lines
Source: Front Microbiol. 2019 Jun 20;10:1432. doi: 10.3389/fmicb.2019.01432 (PMC6596324; doi:10.3389/fmicb.2019.01432)
Supplement: Supplementary file 1 [file Data_Sheet_1.docx]

**Innate Immune Genes Associated with Newcastle Disease Virus Load in Chick Embryos from Inbred and Outbred Lines**

Megan A. Schilling^123^, Sahar Memari^2^, Isabella M. Cattadori^24^, Robab Katani^24^, Amandus P. Muhairwa^6^, Joram J. Buza^3^, Vivek Kapur^1234*^

^1^ Animal Science Department, Pennsylvania State University, University Park, PA

^2^ Huck Institutes of the Life Sciences, Pennsylvania State University, University Park, PA

^3^ School of Life Sciences and Bioengineering, The Nelson Mandela African Institution of Science and Technology, Arusha, TZ

^4^ Applied Biological and Biosecurity Research Laboratory, Pennsylvania State University, University Park, PA

^5^ Department of Biology, Pennsylvania State University, University Park, PA

^6^ Department of Veterinary Medicine and Public Health, Sokoine University of Agriculture, Morogoro, TZ

***Correspondence:**

Vivek Kapur

[vkapur@psu.edu](mailto:vkapur@psu.edu)

**Supplementary Information**

*Supplemental Figures*

*
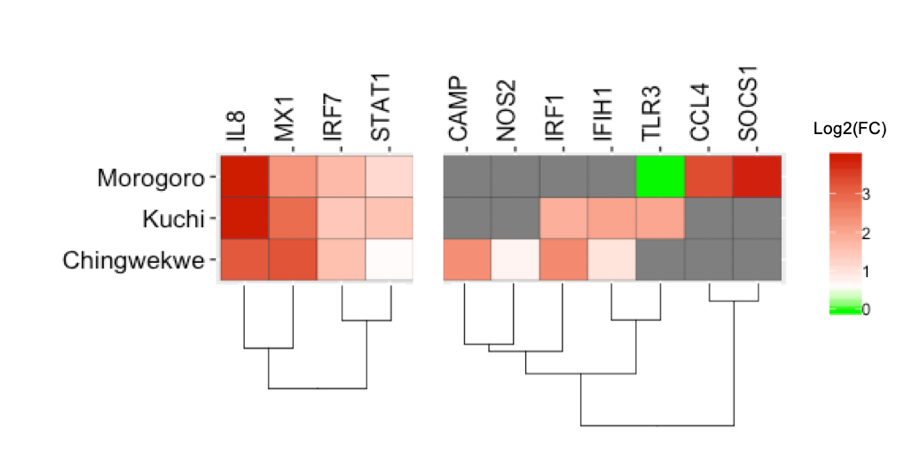
*

**Supplemental Figure 1. Heatmap and Hierarchical Clustering of the Gene Expression Profiles of the Three Ecotypes.** The average log2(Fold Change) expression of each gene is visualized in the heatmap. The red represents genes that are upregulated, the green represents genes that are downregulated. The gray color represents genes that are not significantly expressed compared to the controls within that respective subline. Hierarchical clustering also demonstrates the clustering of conserved genes (differentially expressed over the controls in all ecotypes) and genes that are differentially regulated between the ecotypes.

**
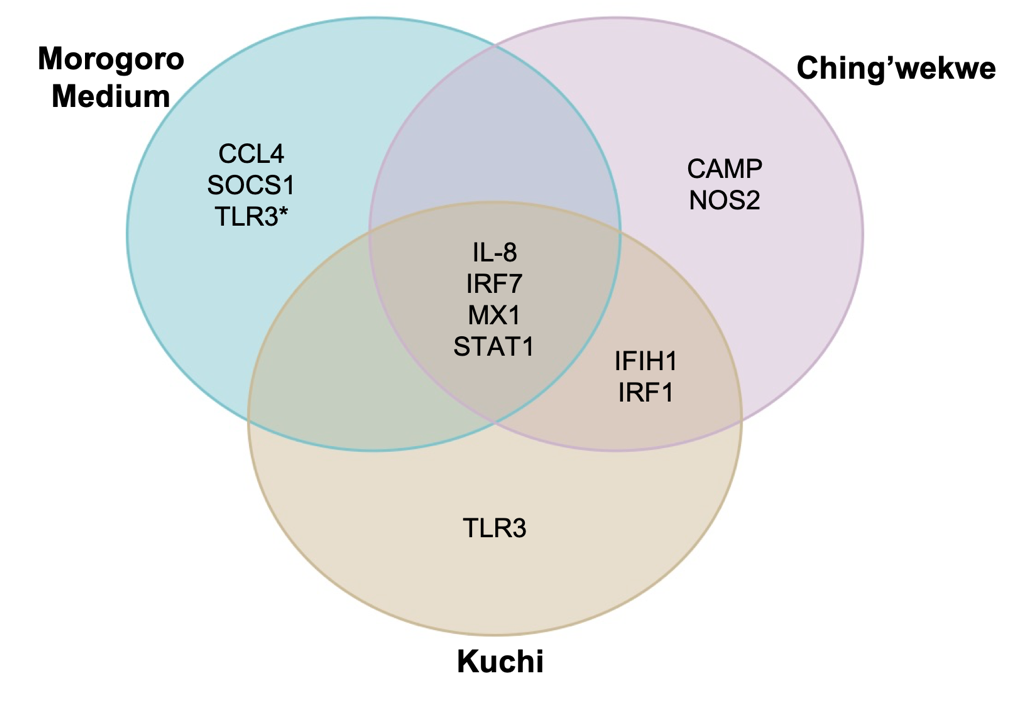
**

**Supplemental Figure 2.** **Venn Diagram representing the genes that are differentially expressed over the controls in each subline**. The Venn Diagram represents the same genes that are differentially expressed in the experimental groups over the controls that are in Supplemental Figure 1. It demonstrates a conserved response of 4 genes in the middle and genes respective to each subline. TLR3 is the only gene that is downregulated, denoted by the *.


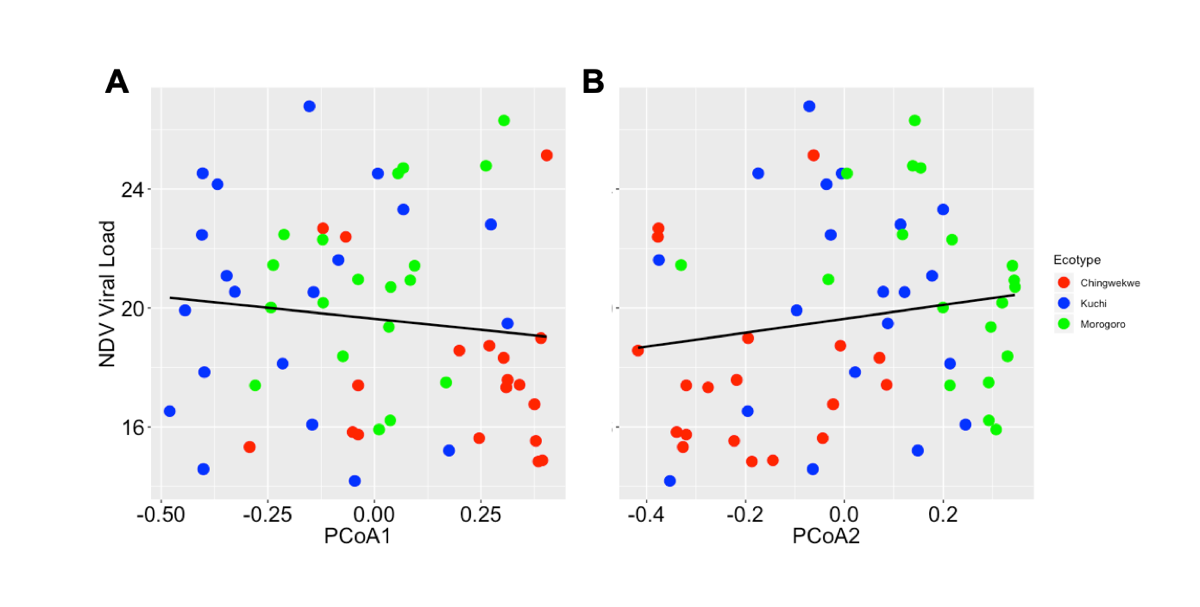


**Supplemental Figure 3. Relationship between principal coordinate (PCoA) axis and NDV viral load.**  **(A)** The NDV viral load (log(copy number)) versus the PCoA axis 1 (PCoA1: 0.11 ± 0.25, p-value = 0.39). The expression of NOS2 is most representative for PCoA axis 1. **(B)** The NDV viral load (log(copy number)) versus the PCoA axis 2 (PCoA2: -0.16 ± 0.36, p-value = 0.23). The expression of CCL4, CAMP, and IL8 are most representative for PCoA axis 2.

**
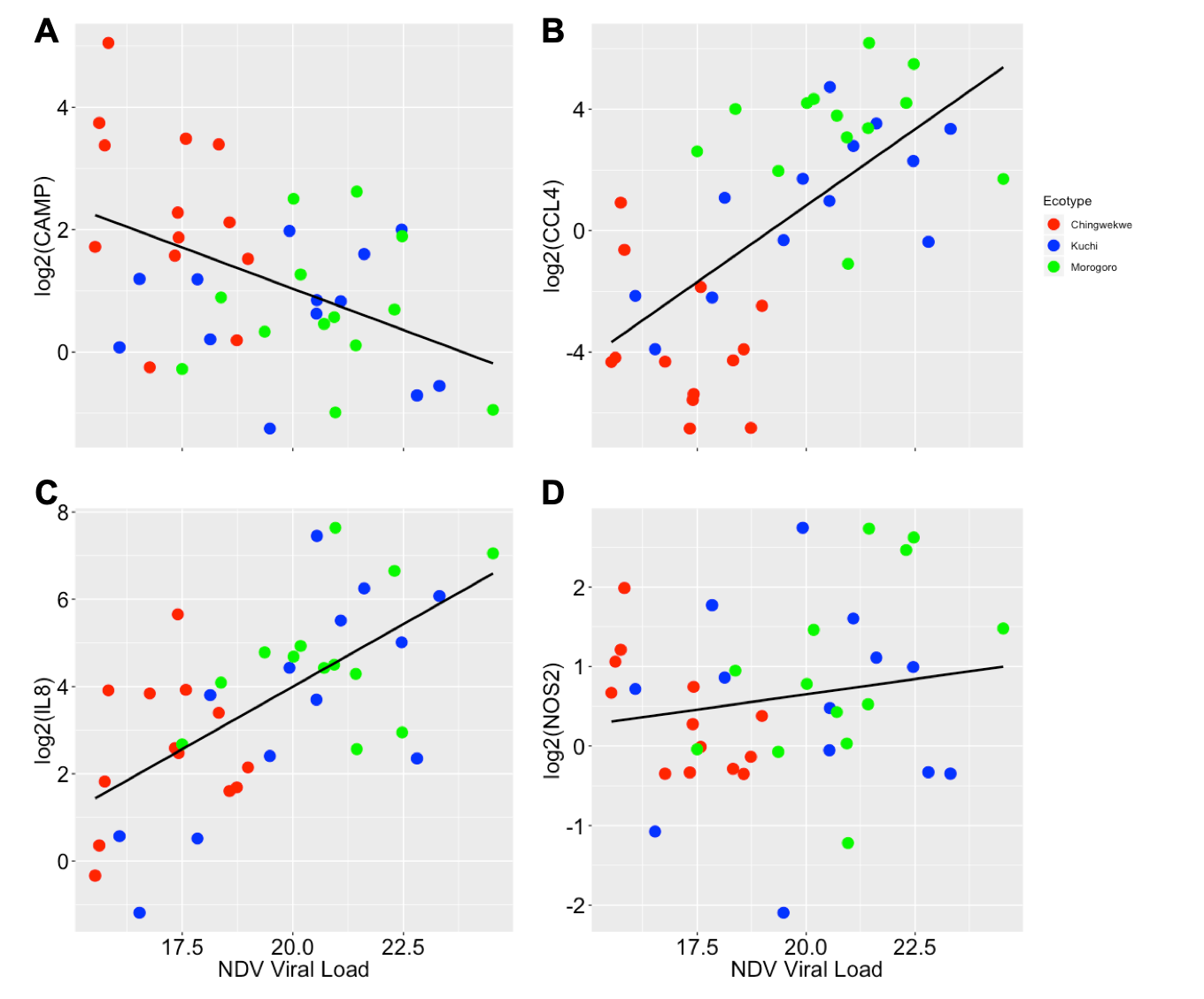
**

**Supplemental Figure 4. Relationship between expression of individual innate immune gene and NDV viral load. (A)** The NDV viral load (log(copy number)) versus CAMP expression (0.436 ± 0.22, p-value < 0.01). **(B)** versus CCL4 expression ((-0.66 ± 0.22, p-value < 0.01). **(C)** versus NOS2 expression (-0.23 ± 0.32, p-value = 0.15). **(D)** versus IL8 expression (-0.667 ± 0.22, p-value < 0.01). Ct values and overall viral load values are inversely related.


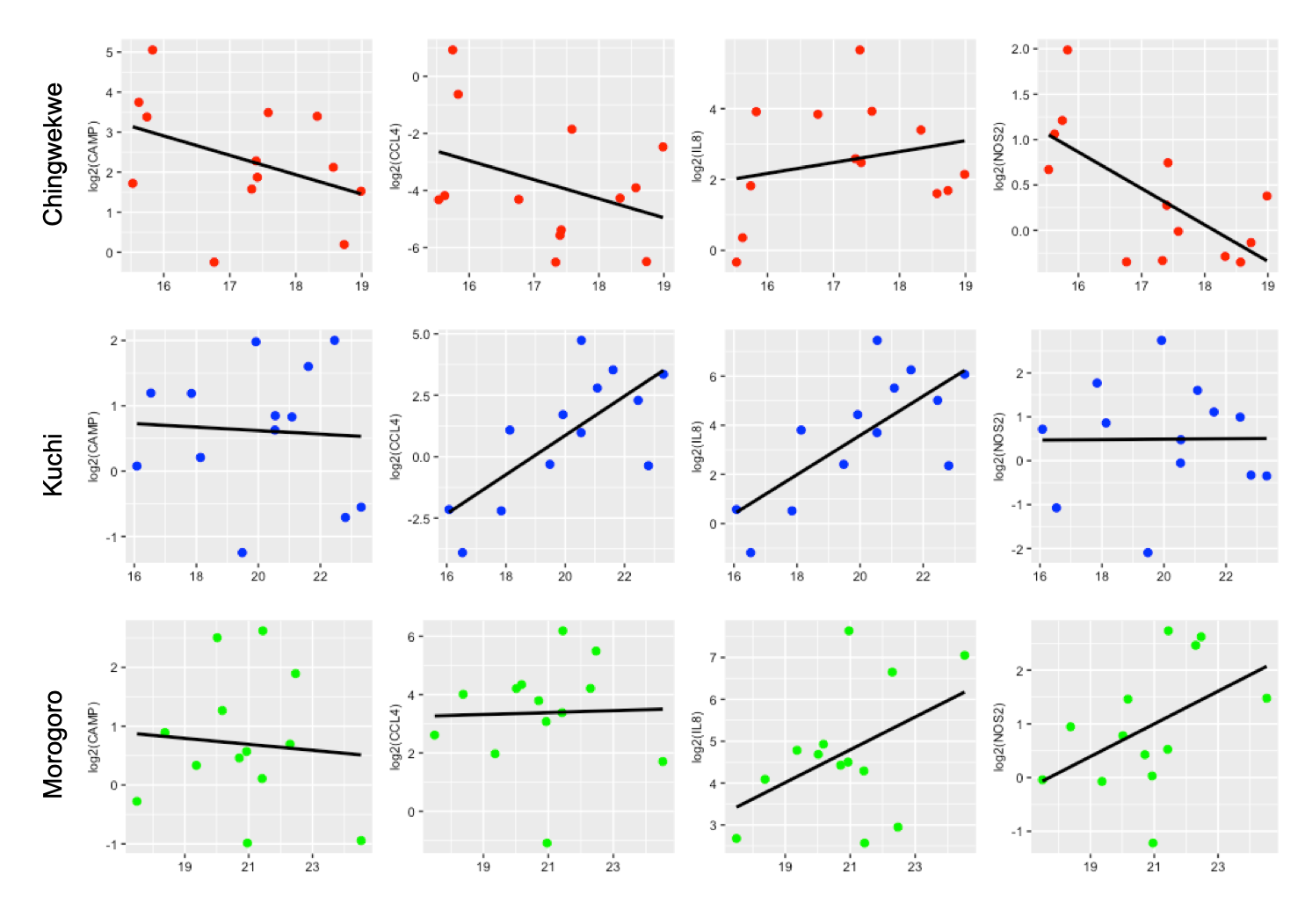


**Supplemental Figure 5. Relationship between expression of individual innate immune gene and NDV viral load within each Ecotype.** The NDV viral load (log(Copy Number)) was plotted versus the log2(Fold Change) of each target. The top row of graphs are the Ching’wekwe, colored in red. The middle row are the Kuchi, colored in blue. The bottom row is the Morogoro Medium, colored in green.


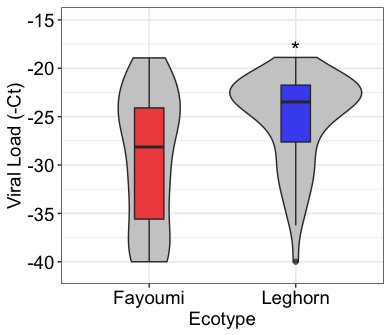


**Supplemental Figure 6. Viral Load of Newcastle Disease Virus in the Chick Embryo Lung Tissues of Fayoumi and Leghorn lines.** The viral load of NDV was assessed two breeds (Fayoumi and Leghorn) along the x-axis and the Ct value from the RT-PCR reaction on the y-axis. The higher the Ct value the lower the viral load and vice versa. The viral load in the Fayoumi is significantly lower than the Leghorn (p-value < 0.01). Within the boxplots, the middle line - median, box - Q1-Q3, whiskers - range, dots – outliers. The violin plots show the distribution of samples. The Ching’wekwe has a significantly lower viral load than the other two ecotypes (p-value < 0.05).

*Supplemental Tables*

**Supplemental Table 1. The targets and assay IDs of the genes examined in the Tanzanian Ecotypes.** The Taqman Assays are available through Thermo Fisher Scientific**.**

| **Target** | **Assay ID** | **Probe Label** | **Reaction** |
| --- | --- | --- | --- |
| ACTB | Gg03815934_s1 | VIC-MGB | 1, 2, 3, 4 |
| MX1 | Gg03337834_m1 | FAM-MGB | 1 |
| IRF1 | Gg03339950_m1 | ABY-QSY | 1 |
| IRF7 | Gg03339759_m1 | JUN-QSY | 1 |
| STAT1 | Gg03343400_m1 | FAM-MGB | 2 |
| SOCS1 | Gg03811978_s1 | ABY-QSY | 2 |
| NOS2 | Gg03347749_m1 | JUN-QSY | 2 |
| IFIH1 | Gg03314321_m1 | FAM-MGB | 3 |
| LITAF | Gg03364359_m1 | ABY-QSY | 3 |
| TLR3 | TRP_qsy | JUN-QSY | 3 |
| IL8L2 | Gg03349360_m1 | FAM-MGB | 4 |
| CCL4 | Gg03338617_m1 | ABY-QSY | 4 |
| CAMP | Gg03344087_m1 | JUN-QSY | 4 |

**Supplemental Table 2. Gene expression values (log2(Fold Change)) for each gene and ecotype from Figure 2.** The table represents the log2(Fold Change) for each target in each subline. This is the data used to generate Figure 2.

| **Target** | **Chingwekwe** | **Kuchi** | **Morogoro** |
| --- | --- | --- | --- |
| IRF1 | 2.435 | 1.830 | 0.938 |
| IRF7 | 1.546 | 1.436 | 1.653 |
| MX1 | 3.244 | 2.877 | 2.253 |
| NOS2 | 0.706 | 0.747 | 0.813 |
| SOCS1 | 1.256 | 4.154 | 3.809 |
| STAT1 | 0.600 | 1.5114 | 1.145 |
| IFIH1 | 0.977 | 2.002 | -0.128 |
| LITAF | 0.890 | -0.176 | -0.617 |
| TLR3 | -0.851 | 1.973 | -0.037 |
| CAMP | 2.348 | 0.836 | 0.563 |
| CCL4 | -1.83 | 1.216 | 3.340 |
| IL8L2 | 3.162 | 3.935 | 3.956 |

**Supplemental Table 3. Range of Viral Load associated with Q1-Q3 in Figure 4.** The corresponding Viral Load (Copy Number) and sample size for the range of values from the first through third quartiles for each ecotype is represented in the table.

| **Ecotype** | **n (Q1-Q3)** | **Viral Load (Copy No.)** |
| --- | --- | --- |
| Ching’wekwe | 13 | 5.5 x 10^6^ – 1.8 x 10^8^ |
| Kuchi | 14 | 9.9 x 10^6^ – 1.3 x 10^10^ |
| Morogoro Medium | 13 | 3.9 x 10^7^ – 4.5 x 10^10^ |
